# Na⁺/Mg²⁺ ratio: a new physiological trait for salt resistance in faba bean (*Vicia faba* L.)

**DOI:** 10.1186/s12870-025-07698-x

**Published:** 2025-11-14

**Authors:** Divya Parisa, Amit Sagervanshi, Muna Ali Abdalla, Karl-Hermann Mühling

**Affiliations:** Institute of Plant Nutrition and Soil Science, Kiel University, Hermann- Rodewald−Str. 2, Kiel, 24118 Germany

**Keywords:** *Vicia faba*, Salinity, Magnesium, Na^+^/Mg²^+^ ratio

## Abstract

Magnesium plays a vital role in enhancing plant resilience under salt stress. However, its specific function in maintaining ion homeostasis, particularly in regulating sodium uptake, remains unclear. Recognizing that magnesium deficiency leads to increased potassium uptake and accumulation, and given that sodium and potassium possess the same charge, we hypothesize that salt stress disrupts ion homeostasis to a greater extent in magnesium-deficient plants compared to those deficient in potassium. To test this hypothesis, *Vicia faba* plants were cultivated hydroponically and subjected to moderate salinity stress (50 mM NaCl) for 2 weeks starting from four weeks after transplanting. The plants were grown under varying levels of magnesium (0.5 mM sufficient; 0.02 mM deficient) and potassium (2 mM sufficient; 0.3 mM deficient), with harvesting occurring two weeks after exposure to salinity. The results indicated that under salinity conditions, magnesium deficiency had a more severe adverse effects on plant growth and gas exchange parameters than potassium deficiency. Stomatal movement was notably restricted in magnesium-deficient plants, potentially due to the over accumulation of soluble sugars and chloride. In magnesium-deficient plants the Na^+^/Mg^2+^ ratio was significantly higher in leaves (17-fold) and in roots (14-fold) relative to Mg^2+^ sufficient plants under salinity stress. Furthermore, the higher K^+^/Mg^2+^ ratio in magnesium-deficient conditions, observed under both saline and non-saline environments, suggests that potassium’s antagonistic effect remains unchanged even under stress conditions. Our findings emphasize for the first time that magnesium, rather than potassium, serves a crucial function in regulating the ion homeostasis necessary for normal plant growth and development in saline environments.

## Introduction

Abiotic stress factors such as heavy metals [1], salinity [2], and drought [3] can cause significant yield and quality losses in plants. These abiotic constraints not only disrupt plant water and nutrient balance but also induce oxidative stress, impair membrane integrity, and alter enzymatic antioxidant systems [4]. Salinity has emerged as a major global threat to agricultural production, with projections indicating that up to 50% of arable land could be lost by 2050 [5]. It leads to two main types of stress in plants, including osmotic and ionic stresses [6–9]. Osmotic stress results from diminished soil water potential, which constrains water accessibility and hinders root uptake. Ionic stress, on the other hand, results from the accumulation of toxic ions such as sodium (Na⁺) and chloride (Cl⁻) in plant tissues, leading to ion toxicity, nutritional disorders, metabolic disruptions, and oxidative stress [10].

Elevated levels of Na⁺ and Cl⁻ impair ionic homeostasis due to impaired uptake and translocation of calcium (Ca²⁺), magnesium (Mg²⁺), phosphate (PO₄³⁻), potassium (K⁺), manganese (Mn²⁺), and iron (Fe^2+^) [11, 12]. This nutrient imbalance impairs critical metabolic processes, including enzyme activities, photosynthesis, and ionic homeostasis. Among these essential nutrients, K⁺ and Mg^2^⁺ are particularly significant for their roles in plant stress tolerance and phloem loading. Various studies have reported the significance of K⁺ in regulating numerous physiological functions, including photosynthesis, osmoregulation, carbohydrate transport, and protein synthesis [13, 14].

Mg²⁺, although often overshadowed by K⁺ in ion homeostasis and osmoregulation, is equally essential for many physiological functions. The interaction between K⁺ and Mg²⁺ under salinity stress is complex and involves multiple factors. Salinity-induced Na⁺ accumulation often antagonizes the uptake of K⁺ and Mg²⁺, disrupting their balance within plant tissues [15]. High K⁺ levels in the soil can indeed cause Mg²⁺ deficiency, even when Mg²⁺ is moderately available, highlighting the antagonistic relationship between the two elements [16]. Mg²⁺ deficiency results in increased K⁺ accumulation, which is suggested due to direct competitive antagonism between K⁺ and Mg²⁺ for the same root transporters [17]. A recent report found that under Mg²⁺ deficiency in oat plants, there is increased accumulation or uptake of K⁺. This suggests a complex interaction between the two elements, with an antagonistic relationship during root uptake and a synergistic interaction during translocation [18, 19].

Faba bean, a leguminous crop of high nutritional and economic value, is particularly sensitive to salinity stress [20], making it an ideal model plant for studying ion interactions (e.g., Mg^2+^ and K^+^) at physiological and biochemical processes under adverse conditions. Numerous studies have emphasized the importance of K^+^ in maintaining ionic and osmotic balance under salinity stress [21]. In contrast, reports about the role of Mg²⁺ in maintaining ion homeostasis, especially under K^+^ and Mg²⁺ interaction coupled with salinity stress, are limited. Thus, providing insights into optimizing nutrient management strategies for improved crop resilience and productivity. Accordingly, the authors aim to test the following two hypotheses: (1) K⁺ and Na⁺ have the same charge, and are thought to compete for the same uptake pathways. Therefore, if a primary/secondary pathway for K⁺ is favoured (e.g., due to Mg²⁺ deficiency), Na⁺ uptake might increase. Thus (2), Mg²⁺ and not K^+^ maintain ion homeostasis and limit Na⁺ uptake under salinity stress.

## Results

### Plant biomass

**Fig. 1:**
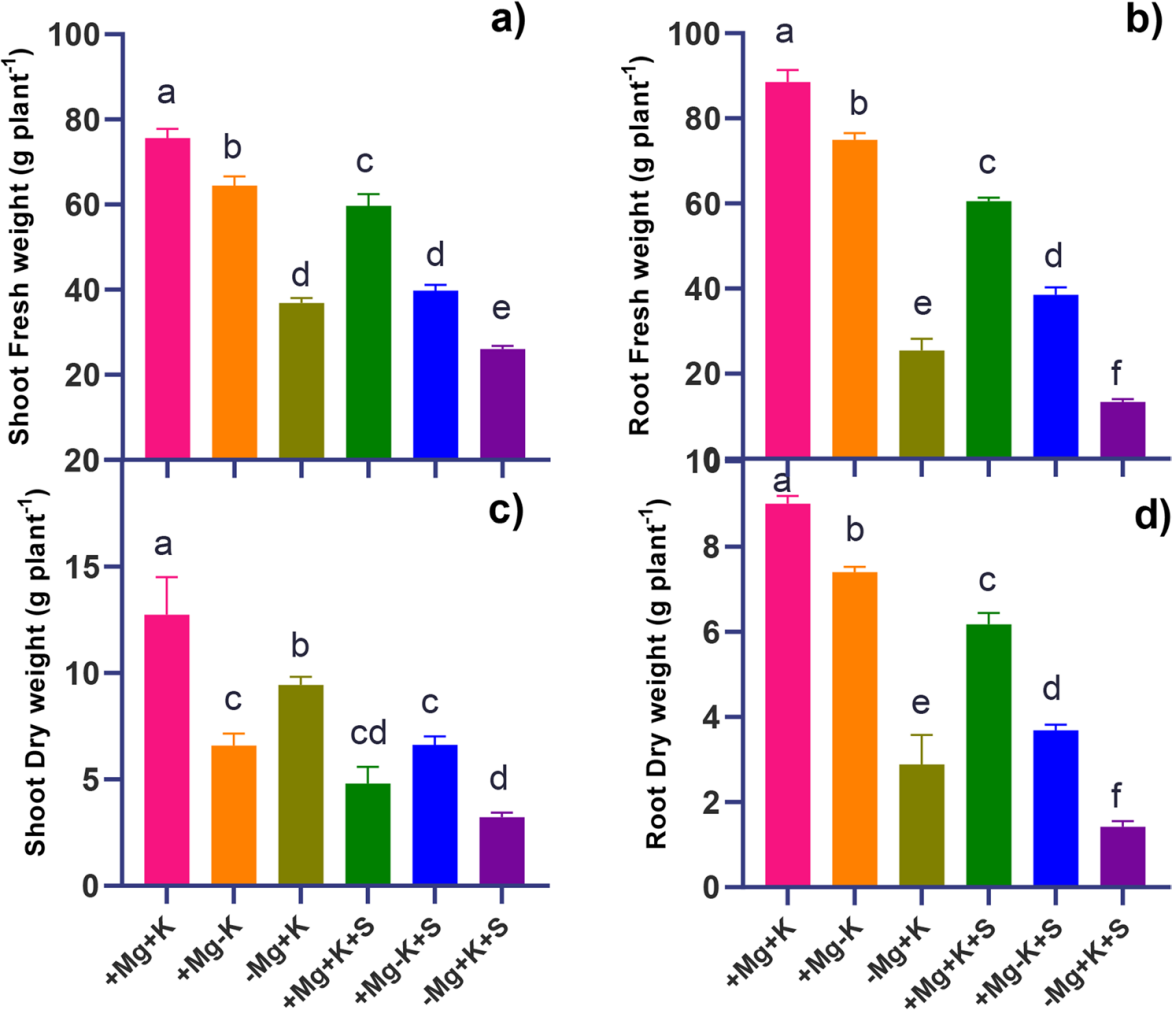
The effect of salinity stress at contrasting levels of Mg^2+^ and K^+^ on the morphological growth parameters in faba bean plants (*Vicia faba* L.) at 45 DAT. Data expressed as **a** SFW **b** RFW **c** SDW **d** RDW. + Mg + K (0.5 mM MgSO_4_ × 2 mM K_2_SO_4_), +Mg − K (0.5 mM MgSO_4_ × 0.3 mM K_2_SO_4_), −Mg + K (0.02 mM MgSO_4_ × 2 mM K_2_SO_4_), + Mg + K + S (0.5 mM MgSO_4_ × 2 mM K_2_SO_4_ × 50 mM NaCl), +Mg − K + S (0.5 mM MgSO_4_ × 0.3 mM K_2_SO_4_ × 50 mM NaCl) and − Mg + K + S (0.02 mM MgSO_4_ × 2 mM K_2_SO_4_ × 50 mM NaCl). Each value is the mean of four replicates; the bars represent ± standard error. Lowercase letters indicate significant differences between treatments (ANOVA with Tukey’s HSD, *p* *≤* 0.05)

Fresh weight (FW) and dry weight (DW) data of the faba bean plants subjected to salinity stress at various levels of Mg^2+^ and K^+^ are presented in Fig. 1. Control plants (+ Mg + K) had reported a higher SFW, with a 21% reduction in SFW when subjected to salinity stress. Mg^2+^ deficiency (− Mg + K), resulted in a 51% reduction in SFW, and salinity stress under Mg^2+^ deficiency (− Mg + K + S) led to a 66% reduction in SFW. Whereas, Mg^2+^ deficiency (MGD) (− Mg + K) has resulted in a reduction of root fresh weight (RFW) by around 71%, and salinity stress (− Mg + K + S) further aggravated RFW reduction with a decrease of about 85%. (Fig. 1). Mg^2+^ deficiency (− Mg + K) has resulted in a 25% decrease in the shoot dry weight (SDW). Meanwhile, similar treatment interaction under salinity (− Mg + K + S) has resulted in a 75% decrease in SDW. K^+^ deficiency (+ Mg − K) resulted in 48% reduction of SDW. Whereas, the same treatment interaction had no changes in SDW under salinity stress. A significant reduction in RDW to 68% and 84% was recorded in the treatment interaction of deficient Mg^2+^ and sufficient K^+^ (− Mg + K), both under control conditions as well as salinity stress (− Mg + K + S), respectively (Fig. 1).

### SPAD, gas exchange, osmolyte accumulation and stomatal movements

**Fig. 2:**
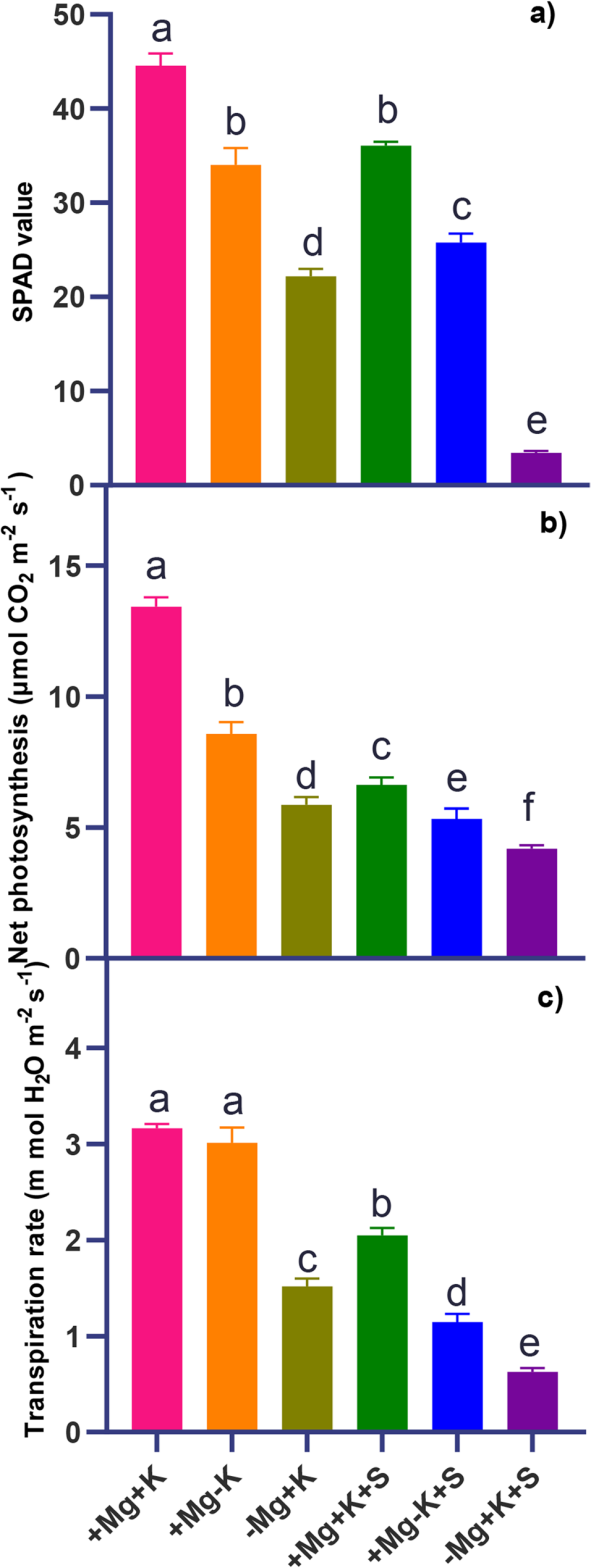
**a** Course of SPAD values of the fourth leaf **b** Net CO_2_ assimilation rate (µmol CO_2_ m^− 2^ s^− 1^) of the fourth leaf, and **c** transpiration rate mmol H_2_O m^− 2^s^− 1^ of the fourth leaf. + Mg + K (0.5 mM MgSO_4_ × 2 mM K_2_SO_4_), +Mg − K (0.5 mM MgSO_4_ × 0.3 mM K_2_SO_4_), −Mg + K (0.02 mM MgSO_4_ × 2 mM K_2_SO_4_), + Mg + K + S (0.5 mM MgSO_4_ × 2 mM K_2_SO_4_ × 50 mM NaCl), +Mg − K + S (0.5 mM MgSO_4_ × 0.3 mM K_2_SO_4_ × 50 mM NaCl) and − Mg + K + S (0.02 mM MgSO_4_ × 2 mM K_2_SO_4_ × 50 mM NaCl). Each value is the mean of four replicates; the bars represent ± standard error. Lowercase letters indicate significant differences between treatments (ANOVA with Tukey’s HSD, *p* *≤* 0.05)

MGD symptoms (interveinal chlorosis) on the older leaves of faba bean plants grown under Mg^2+^ limitation started after three weeks from transplanting. Consequently, K^+^ deficiency symptoms (yellowing of the leaf margins and browning of the leaf edges) aggravated the severity of these symptoms under both deficiencies of Mg^2+^ and K^+^. As expected, the Chl. values were significantly reduced (by 92%) in Mg^2+^ deficient plants under salinity (− Mg + K + S) (Fig. 2a). Gas exchange measurements revealed significant differences in net CO₂ assimilation and transpiration rates of the fourth leaf across all treatment interactions (Fig. 2b and c). Control plants (+ Mg + K) have exhibited stable assimilation rates of around 15 µmol CO_2_ m^− 2^s^− 1^. In contrast, plants grown under − Mg + K + S had a 68% reduction in net photosynthesis, coupled with an 80% reduction in transpiration rate, compared to the control. This reduction had almost reached zero by 15 days after salinity initiation, as the plants began to wither and wilt. On the other hand, plants under salt stress (+ Mg + K + S) with sufficient Mg^2+^ and K^+^ displayed a 53% reduction in net photosynthesis and a 35% reduction in transpiration rate compared to the control (+ Mg + K).

**Fig. 3:**
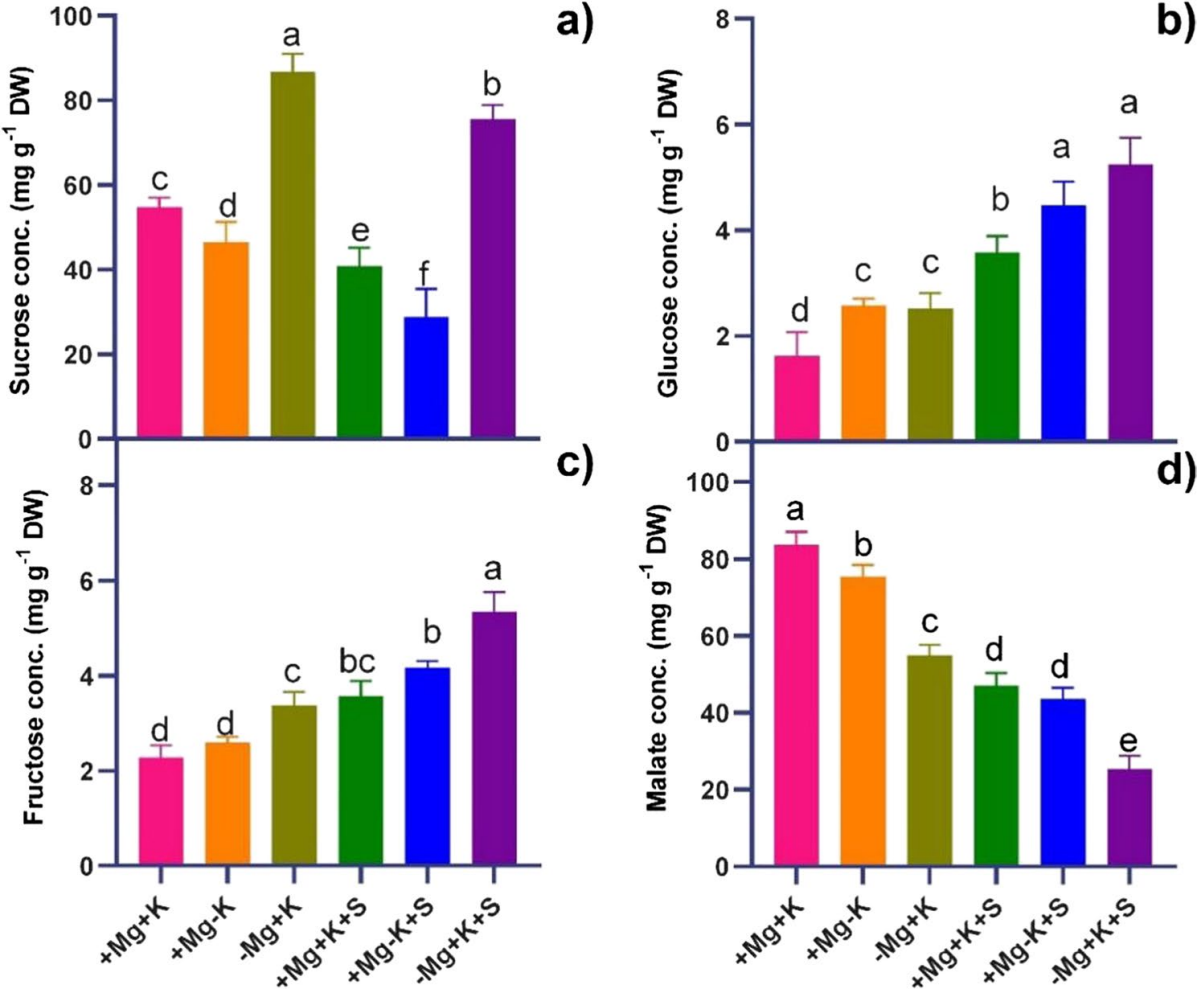
Accumulation of osmolytes: **a** Sucrose; **b** Glucose, **c** Fructose, and **d** Malate concentrations (conc.) per mg g^− 1^ DW in leaves harvested at 45 DAT. + Mg + K (0.5 mM MgSO_4_ × 2 mM K_2_SO_4_), +Mg − K (0.5 mM MgSO_4_ × 0.3 mM K_2_SO_4_), −Mg + K (0.02 mM MgSO_4_ × 2 mM K_2_SO_4_), + Mg + K + S (0.5 mM MgSO_4_ × 2 mM K_2_SO_4_ × 50 mM NaCl), +Mg − K + S (0.5 mM MgSO_4_ × 0.3 mM K_2_SO_4_ × 50 mM NaCl) and − Mg + K + S (0.02 mM MgSO_4_ × 2 mM K_2_SO_4_ × 50 mM NaCl). Each value is the mean of four replicates; the bars represent ± standard error. Lowercase letters indicate significant differences between treatments (ANOVA with Tukey’s HSD, *p* *≤* 0.05)

MGD triggered sucrose accumulation in the leaves of − Mg + K (57%) and − Mg + K + S (45%) (Fig. 3a). Hexose sugars Glu and Fru concentrations were significantly higher under − Mg + K + S treatment, with a 2 − fold increase in Fru concentration and a 4 − fold increase in Glu concentration compared to control plants (+ Mg + K) (Fig. 3b, c). MGD (− Mg + K) decreased malate by 37% compared to control (+ Mg + K). Meanwhile, MGD under salinity stress (− Mg + K + S) further declined malate by 68% when compared to the control (+ Mg + K) (Fig. 3d).

**Fig. 4:**
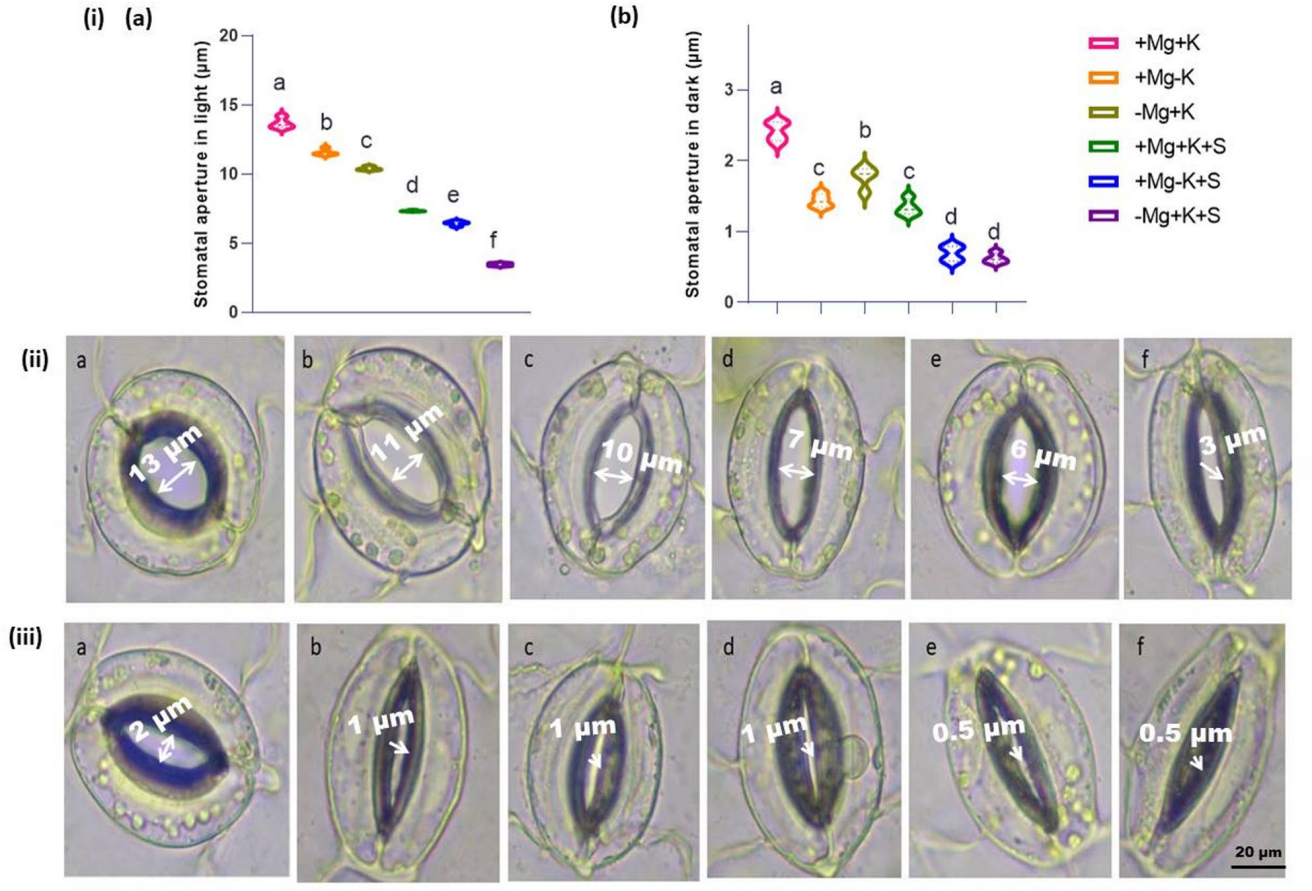
**i** Analysis of stomatal apertures under light microscope **i.a** Stomata opening assay and **i.b** Stomata closing assay. **ii** Analysis of stomata opening in response to light (scale bar 20 μm) **iii** stomata closure in response to dark (scale bar 20 μm). The arrows indicate the aperture width of the guard cells. **ii**&**iii** **a** + Mg + K (0.5 mM MgSO_4_ × 2 mM K_2_SO_4_), **b** + Mg − K (0.5 mM MgSO_4_ × 0.3 mM K_2_SO_4_), **c** − Mg + K (0.02 mM MgSO_4_ × 2 mM K_2_SO_4_), **d** + Mg + K + S (0.5 mM MgSO_4_ × 2 mM K_2_SO_4_ × 50 mM NaCl), **e** + Mg − K + S (0.5 mM MgSO_4_ × 0.3 mM K_2_SO_4_ × 50 mM NaCl) and **f** − Mg + K + S (0.02 mM MgSO_4_ × 2 mM K_2_SO_4_ × 50 mM NaCl). The inner width of the stomatal pore was measured, and four replicates were used for each treatment. Each value is the mean of four replicates; Lowercase letters indicate significant differences between treatments (ANOVA with Tukey’s HSD, *p* *≤* 0.05)

In agreement with the reduced transpiration rates, the stomatal apertures were reduced in − Mg + K + S plants under light after 2 weeks of NaCl stress compared to the control (Fig. 4i.a). However, the aperture of the stomatal pores of leaves of various treatment interactions was differently regulated because the salt stress − induced reduction was dependent on Mg^2+^ and K^+^ concentrations. Moreover, young leaves of control plants (+ Mg + K) had widely opened stomata, whereas the apertures of the same treatment interaction were reduced by 46% under NaCl conditions. In contrast to the differential aperture regulation in light, the stomata of MGD plants (− Mg + K + S) were reduced by 75% compared to the control (Fig. 4 (ii.f)). Under dark-induced closing stimuli, −Mg + K + S plants showed a stronger response than controls, reducing stomatal aperture width by 74% compared to untreated plants (Fig. 4 (iii.f)).

### Ion concentration in leaves

**Fig. 5:**
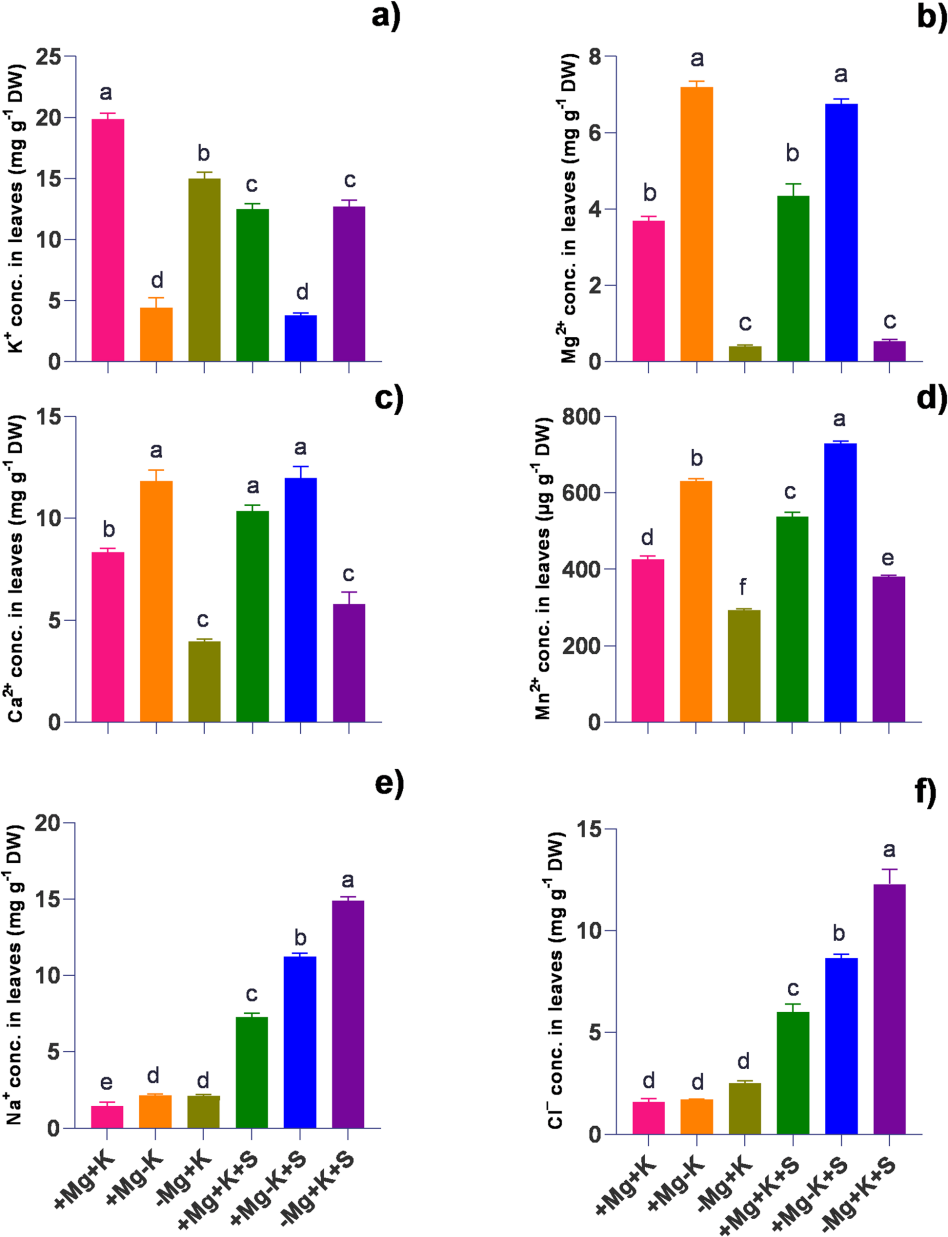
Effect of varied Mg^2+^ and K^+^ supply on macro and micronutrient and Na^+^ Cl^−^ concentration (Conc.) in leaves harvested at 45 DAT. **a **K^+^ conc. in leaves (mg g^-1^ DW) **b** Mg^2+^ conc. in laves (mg g^-1^ DW) **c** Ca^2+^ conc. in leaves (mg g^-1^ DW) **d** Mn^2+^ conc. in leaves (µg g^-1^ DW) **e** Na^+^ conc. in leaves (mg g^-1^ DW) **f** Cl^-^ conc. in leaves (mg g^-1^ DW). + Mg + K (0.5 mM MgSO_4_ × 2 mM K_2_SO_4_), +Mg − K (0.5 mM MgSO_4_ × 0.3 mM K_2_SO_4_), −Mg + K (0.02 mM MgSO_4_ × 2 mM K_2_SO_4_), + Mg + K + S (0.5 mM MgSO_4_ × 2 mM K_2_SO_4_ × 50 mM NaCl), +Mg − K + S (0.5 mM MgSO_4_ × 0.3 mM K_2_SO_4_ × 50 mM NaCl) and − Mg + K + S (0.02 mM MgSO_4_ × 2 mM K_2_SO_4_ × 50 mM NaCl). Values represent the mean of four replicates ± standard error. Lowercase letters indicate significant differences between treatments (ANOVA with Tukey’s HSD, *p* *≤* 0.05)

A higher amount of K^+^ content was observed under control conditions (+ Mg + K) with sufficient Mg^2+^ and K^+^. In contrast, under salinity, K^+^ levels were found to be similar in + Mg + K + S and − Mg + K + S plants, with a decrease of 37% and 36%, respectively, compared to the control plants. In contrast, K⁺ levels were significantly reduced under both K-deficient (+ Mg–K) and salinity-stressed (+ Mg–K + S) treatments, with total reductions of 78% and 81%, respectively. Under control conditions, a significantly lower amount of Ca^2+^ was found in Mg^2+^ deficient plants (− Mg + K). K^+^ deficiency resulted in higher accumulation (by 42% and 44%) of Ca^2+^ both under control (+ Mg − K) conditions as well as under salinity stress (+ Mg − K + S). A significantly higher amount of Mg^2+^ was noticed in Mg^2+^ sufficient and K^+^ deficient (+ Mg − K) conditions, with a 2 − fold increase and 95% increase in Mg^2+^ concentration compared to the control (+ Mg + K). Salinity stress under similar conditions of sufficient Mg^2+^ and deficient K^+^ (+ Mg − K + S) results in an 83% increase in Mg^2+^ concentration compared to the control. A higher amount of Mn^2+^ (1.5 − fold) was recorded under K^+^ deficient conditions (+ Mg − K). Salinity stress (+ Mg − K + S) resulted in a higher accumulation of Mn^2+,^ with a 71% increase compared to control conditions. The addition of 50 mM NaCl resulted in the accumulation of Na^+^ and Cl^−^ (Fig. 5e, and f). A significant increase of 10 − fold and 8 − fold, in Na^+^ and Cl^−^, respectively, was noticed under conditions of Mg^2+^ deficiency with sufficient K^+^ levels (− Mg + K + S) under salinity stress. Antagonistic relations of K^+^ on Mg^2+^ uptake, and Mg^2+^ on Na^+^ uptake, and K^+^ on Na^+^ uptake were noticed (Fig. 7i.a., b, and c), with a significantly higher 17 − fold increase in Na^+^/Mg^2+^ in magnesium-deficient plants under salt stress (− Mg + K + S) compared to Mg^2+^ and K^+^ sufficient plants (+ Mg + K + S).

### Ion concentration in roots

**Fig. 6:**
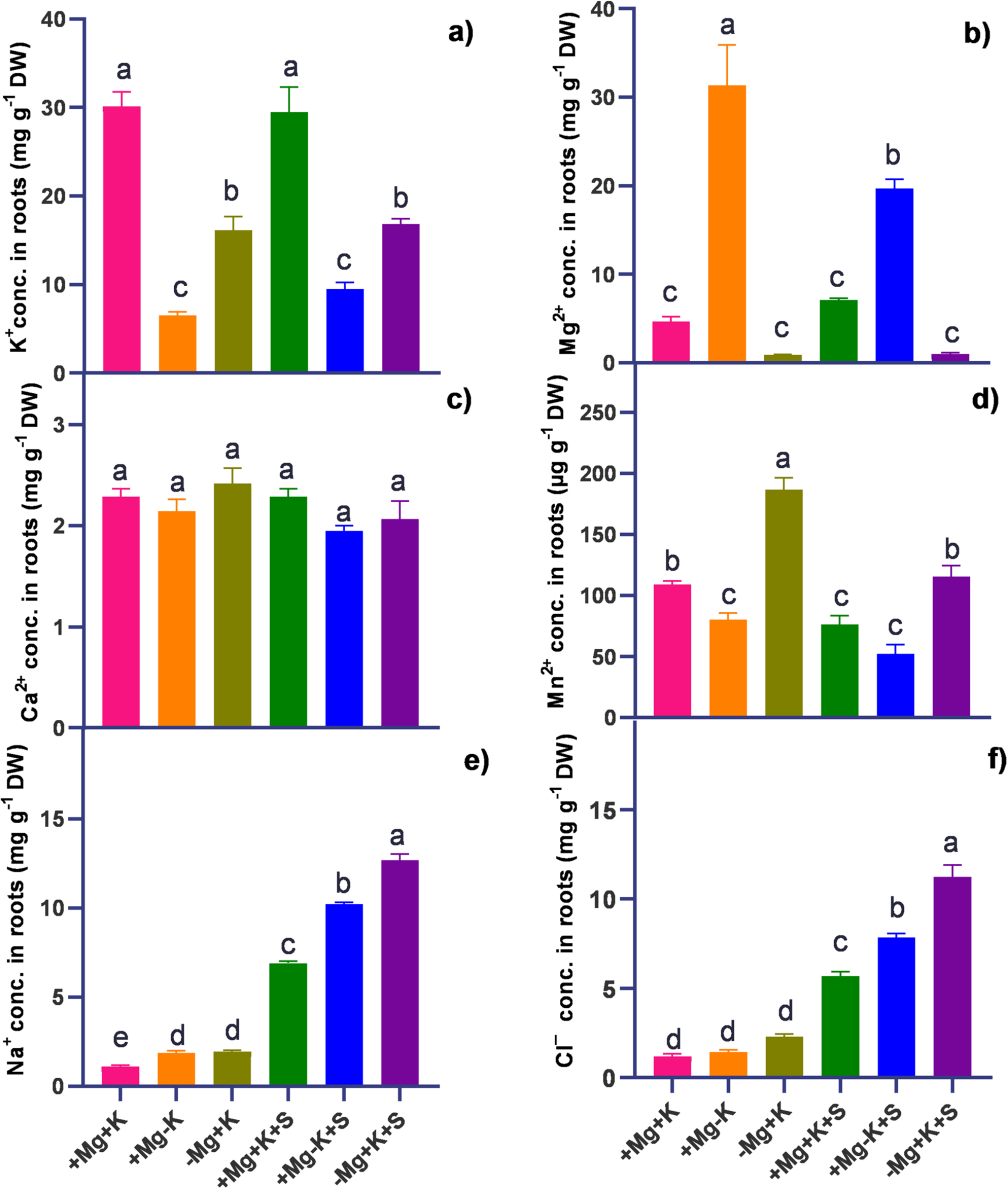
Effect of varied Mg^2+^ and K^+^ supply on macro and micronutrient and Na^+^ Cl^−^ concentration (conc.) in roots harvested at 45 DAT. **a** K^+^ conc. in roots (mg g^-1^ DW) **b** Mg^2+^ conc. in roots (mg g^-1^ DW) **c** Ca^2+^ conc. in roots (mg g^-1^ DW) **d** Mn^2+^ conc. in roots (µg g^-1^ DW) **e** Na^+^ conc. in roots (mg g^-1^ DW) **f** Cl^−^ conc. in roots (mg g^-1^ DW)+ Mg + K (0.5 mM MgSO_4_ × 2 mM K_2_SO_4_), +Mg − K (0.5 mM MgSO_4_ × 0.3 mM K_2_SO_4_), −Mg + K (0.02 mM MgSO_4_ × 2 mM K_2_SO_4_), + Mg + K + S (0.5 mM MgSO_4_ × 2 mM K_2_SO_4_ × 50 mM NaCl), +Mg − K + S (0.5 mM MgSO_4_ × 0.3 mM K_2_SO_4_ × 50 mM NaCl) and − Mg + K + S (0.02 mM MgSO_4_ × 2 mM K_2_SO_4_ × 50 mM NaCl). Values represent the mean of four replicates ± standard error. Lowercase letters indicate significant differences between treatments (ANOVA with Tukey’s HSD, *p* *≤* 0.05)

**Fig. 7:**
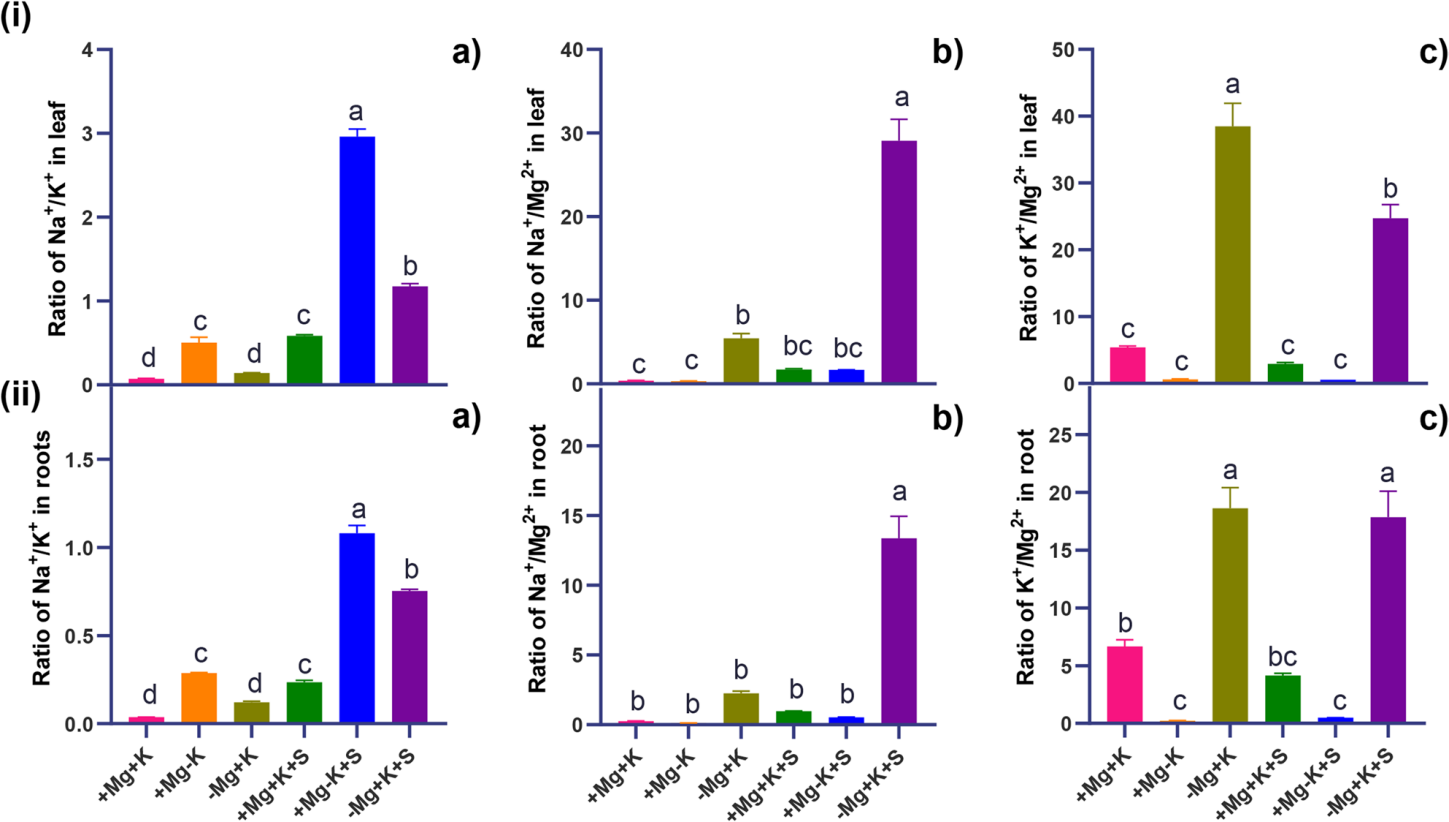
Variability ratio of **i.a** Na^+^/K^+^ in leaves, **i.b** Na^+^/Mg^2+^ in leaves, **i.c** K^+^/Mg^2+^ in leaves, **ii.a** Na^+^/K^+^ in roots, **ii.b** Na^+^/Mg^2+^ in roots, **ii.c** K^+^/Mg^2+^ in roots harvested at 45 DAT. + Mg + K (0.5 mM MgSO_4_ × 2 mM K_2_SO_4_), +Mg − K (0.5 mM MgSO_4_ × 0.3 mM K_2_SO_4_), −Mg + K (0.02 mM MgSO_4_ × 2 mM K_2_SO_4_), + Mg + K + S (0.5 mM MgSO_4_ × 2 mM K_2_SO_4_ × 50 mM NaCl), +Mg − K + S (0.5 mM MgSO_4_ × 0.3 mM K_2_SO_4_ × 50 mM NaCl) and − Mg + K + S (0.02 mM MgSO_4_ × 2 mM K_2_SO_4_ × 50 mM NaCl). Values represent the mean of four replicates ± standard error. Lowercase letters indicate significant differences between treatments (ANOVA with Tukey’s HSD, *p* *≤* 0.05)

A high concentration of K^+^ was found under control conditions (+ Mg + K). Salinity stress had no impact on root K^+^ concentration under similar conditions. On the other hand, K^+^ concentration was found to decrease under salinity stress with sufficient Mg^2+^ and deficient K^+^ (+ Mg − K + S), resulting in a percentage decrease of 68%. Root Ca²⁺ levels remained unchanged, with no significant differences detected under the tested conditions. A significantly higher amount of Mg^2+^ (by 7-fold) was detected under sufficient Mg^2+^ and deficient K^+^ concentrations (+ Mg − K). When subjected to salinity stress, the concentration of Mg^2+^ was 4-fold higher compared to control (+ Mg + K) (Fig. 6b). A significantly higher amount of Mn^2+^ was observed under Mg^2+^ deficient and K^+^ sufficient interaction (− Mg + K). The similar treatment interaction, when subjected to salinity stress, led to a decrease in root Mn^2+^ content. The lower levels of Mn^2+^ were observed under sufficient Mg^2+^ and deficient K^+^ conditions (+ Mg − K and + Mg − K + S), with reductions of 24% and 50%, respectively, compared to control (+ Mg + K). A significantly higher concentration of Na^+^ and Cl^−^ was found under Mg^2+^ deficient and K^+^ sufficient treatment interaction following salinity stress (− Mg + K + S), with 11 − fold and 10 − fold increase in Na^+^ and Cl^−^ concentration, respectively. Antagonistic effect of the ions ratio of K^+^ to Mg^2+^ uptake, and Na^+^ on Mg^2+^ uptake, and Na^+^ on K^+^ uptake was noticed in roots (Fig. 7 ii. a, b, and c), with a significantly highest 14-fold increase in Na^+^/Mg^2+^ in magnesium-deficient plants under salt stress compared to Mg^2+^ and K^+^ sufficient plants under salinity stress (+ Mg + K + S).

## Discussion

Despite extensive research on salinity, the role of magnesium in influencing ion homeostasis beyond its established functions in photosynthesis and enzymatic regulation under saline conditions, remains poorly understood. In this study, we present novel findings highlighting the importance of magnesium ions over potassium ions in maintaining ion homeostasis and reducing sodium uptake in plants subjected to salt stress.

### Magnesium deficiency intensified salinity-induced plant growth reduction

In the present study, salinity stress resulted in a decrease in both shoot and root fresh weight and dry weight across all Mg^2+^ and K^+^ treatment levels, with the highest decrease observed under MGD (Fig. 1). This reduction aligns with the expected stress impact on plant growth, where osmotic and ionic stresses disrupt cellular processes, reduce water uptake, and limit photosynthesis, thereby impairing biomass accumulation. Similar reports were observed in the case of faba bean [22] and bean (*Phaseolus vulgaris*) [23]. A substantial decrease in root biomass under Mg^2+^ deficiency, particularly under stress, indicates impaired root development and function due to disrupted Mg^2+^−dependent enzyme activities essential for cellular growth and division, as highlighted by [24] in potatoes and [25] in faba bean. MGD in plants led to interveinal chlorosis on older leaves (Fig. 1), appearing around three WAT with a decline in chlorophyll content. This chlorosis is consistent with Mg’s role as a central component of the chlorophyll molecule, necessary for chloroplast function and photosynthesis [26]. Faba beans grown under saline conditions respond by reducing transpiration to avoid undesired water loss [27]. Our study similarly noted a reduction in net photosynthesis and transpiration across all three treatments under saline conditions (Fig. 2b and c). Notably, the most significant decrease was observed in magnesium-deficient plants when compared to potassium-deficient plants under the same saline conditions. This phenomenon may be attributed to the over-accumulation of Na^+^ and Cl^−^ (Fig. 5e and f) in the leaves, has resulted in decreased transpiration. MGD under sufficient K^+^ nutrition (− Mg + K and − Mg + K + S) triggered a compensatory increase in osmolytes like soluble sugars (glucose, fructose, and sucrose) [28], especially under salinity. This dramatic rise in soluble sugar under salinity is suggested as a key osmo-protectant when Mg^2+^ is deficient, helping to stabilize cellular structures and protect against osmotic imbalance caused by high salt concentrations. Mesophyll-derived sucrose (Suc), energized by H⁺-ATPase activity, serves as an osmotic agent driving light-induced stomatal opening. While Suc accumulates during the afternoon to promote stomatal aperture, excessive concentrations paradoxically trigger stomatal closure [29]. This dual regulatory role of Suc, promoting either opening or closure depending on its concentration, highlights its complex function in stomatal dynamics. Under salinity stress, a deficiency in malate has been observed [30], indicating potential interactions between carbon metabolism and ionic imbalances in stomatal regulation, which align with our findings (Fig. 3d). Notably, MGD induced a greater reduction in malate compared to K^+^ deficiency under salt stress (Fig. 3d). This effect may be linked to the excessive accumulation of chloride (Fig. 5f), which can substitute for malate in its anionic functions and hinder its synthesis. Furthermore, Mg^2+^ serves as a co-factor in key metabolic enzymes; its absence disrupts the production of critical organic acids such as malate, thereby signaling a decline in metabolic resilience under MGD. In salinity conditions, this shortage of organic acids may hinder the plant’s ability to buffer pH and maintain efficient carbon metabolism, ultimately restricting overall stress tolerance. Malate synthesis typically accompanies stomatal opening, as 50% of the K^+^ absorbed can be balanced by malate [31]. The marked reduction in stomatal aperture (Fig. 4) under extended MGD reflects the combined effects of both salinity stress and MGD. Without adequate Mg^2+^, plants lack essential resources for effective stomatal regulation, which compromises gas exchange and results in decreased photosynthetic activity (Fig. 2b and c) and overall growth (Fig. 1a, and b) under salinity conditions.

### Magnesium modulates ion homeostasis under salt stress

As expected, plants subjected to saline conditions exhibited significant accumulation of Na^+^ and Cl^−^ in both their roots and leaves, with magnesium-deficient plants showing greater accumulation compared to potassium-deficient and control plants (Figs. 5 and 6). Furthermore, the elevated ratio of K^+^/Mg^2+^ (7-fold in leaves and 3-fold in roots under non-saline conditions, and 5-fold in leaves and 3-fold in roots under saline conditions) underscores the pronounced antagonistic effects of K^+^ on Mg^2+^ uptake (Fig. 7i.c., and ii.c). This finding is consistent with the observations made by [32]. An antagonism in root-to-shoot translocation has been documented, particularly among members of the *Poaceae* family, as highlighted by [33] in wheat and [17] in rice. Notably, our current study extends these findings to the *Fabaceae* family of plants under both non-saline and saline conditions.

In this study, salinity under K^+^ deficiency resulted in a 5-fold increase in the Na^+^/K^+^ ratio in leaves and roots (Fig. 7i.a, and ii.a), compared to K^+^ sufficient plants under salt stress suggesting variations in the Na^+^/K^+^ ratio among genotypes correlate with salt resistance. It is well-established that K^+^ deficiency stimulates Na^+^ uptake in plants. However, our study revealed a novel observation that magnesium deficiency under saline conditions significantly increased Na^+^ uptake, as evidenced by a 17-fold increase in the Na^+^/Mg^2+^ ratio in leaves and a 14-fold increase in roots (Fig. 7i.b and ii.b) compared to Mg^2+^ sufficient plants. This effect might be related to magnesium deficiency, induced high − affinity magnesium − specific and unspecific transporters’ expressions [18] involved in higher accumulation or uptake of K⁺ and Na^+^, which supports our current hypothesis. This increase correlates with impaired plant growth, highlighting the critical role of Mg^2+^ in maintaining ion homeostasis in plant leaves.

While looking at other nutrient interactions, Ca^2+^ uptake in roots is less influenced by ionic competition under both nutrient sufficiency and salinity stress, a phenomenon supported by its selective transport pathways [34]. Roots under MGD and K^+^ sufficiency (− Mg + K) showed elevated Mn^2+^ concentrations, reflecting the interplay between Mg^2+^ and Mn^2+^ uptake (Fig. 6d). Mg^2+^ deficiency in roots often triggers enhanced Mn^2+^ uptake as part of a compensatory response [35, 36]. However, under salinity stress, root Mn^2+^ concentrations declined, likely due to ionic antagonism from increased Na^+^ and Cl^−^ concentrations. These findings mirror those of [37], who observed diminished Mn^2+^ availability in saline soils, with significant implications for root function and health under combined stress conditions. The drastic increases in root Na^+^ and Cl^−^ concentrations (by 13 − fold and 11 − fold, respectively) (Fig. 6e, and f) under Mg^2+^ deficiency and K^+^ sufficiency during salinity stress (− Mg + K + S) reveal the susceptibility of root ionic balance to salinity under MGD. This accumulation indicates disrupted selectivity in root ion channels, as MGD reduces the activity of specific transporters, exacerbating Na^+^ and Cl^−^ uptake [10]. Such root − specific ionic disruptions are critical, as they can impair root metabolic functions and reduce overall plant vigor.

## Conclusion

This study is the first to comprehensively document the importance of magnesium nutrition for ion homeostasis, extending beyond its established roles in photosynthesis and enzymatic regulation under salinity. The results obtained under the specified experimental conditions support the hypothesis that the growth of magnesium-deficient plants is more adversely affected than that of potassium-deficient plants in saline environments, primarily due to a prolonged imbalance in ion homeostasis. Notably, the findings underscore the crucial role of magnesium ions in maintaining ion homeostasis and limiting sodium uptake under salt stress. Additionally, the observed higher ratio of K⁺/Mg²⁺ in both leaves and roots of magnesium-deficient plants under non-saline and saline conditions indicates a significant increase in potassium uptake, demonstrating a strong antagonistic relationship with magnesium uptake. Overall, this study emphasizes the Na⁺/Mg²⁺ ratio as a vital diagnostic tool for evaluating salinity-induced damage in crop species.

## Materials and methods

### Plant material and growing conditions

The research was conducted at the Institute of Plant Nutrition and Soil Science, located at Kiel University, Germany, focusing on the *Vicia faba* L. cultivar Mallory, with seeds sourced from Norddeutsche Pflanzenzucht Hans-Georg Lembke KG (NPZ) in Hohenlieth, Germany. The seeds were initially germinated on sandwich blots moistened with a 1:5 dilution of 10 mM CaSO_4_ solution. Once germinated, the seedlings were transplanted into 10-L plastic pots, each containing four plants, and supplied with a nutrient solution that started at one-quarter strength and gradually increased to full concentration. The plants were cultivated in a climate chamber under a 14/10-h photoperiod, with day/night temperatures of 20/16°C, 50/60% humidity and a photosynthetically active radiation (PAR) of 250 µmol m⁻² s⁻¹. The standard nutrient solution is a modified Hoagland nutrient solution comprised 2.0 mM Ca(NO_3_)_2_, 2.0 mM K_2_SO_4_, 0.1 mM NH_4_H_2_PO_4_, 0.2 mM KCl, 0.5 mM MgSO_4_, 10 µM H_3_BO_4_, 2.0 µM MnSO_4_, 0.5 µM ZnSO_4_, 0.2 µM CuSO_4_, 0.05 µM (NH_4_)_6_Mo_7_O_24_, and 60 µM Fe-EDTA. For treatments involving K^+^ or Mg^2+^ deficiency, the concentrations were adjusted to 0.3 mM K_2_SO_4_ and 0.02 mM MgSO_4_, respectively. The total SO₄ in the K-deficient treatments is ~ 0.8 mM, and the total SO₄ in the Mg-deficient treatments is ~ 2.0 mM. Sulphate sufficiency for plants typically ranges from 0.2 to 1.0 mM. 0.8 mM is well within the sufficient range and will not induce a sulphur deficiency.

Plants were subjected to six different treatments i.e. + Mg + K (0.5 mM MgSO_4_ × 2 mM K_2_SO_4_), +Mg − K (0.5 mM MgSO_4_ × 0.3 mM K_2_SO_4_), −Mg + K (0.02 mM MgSO_4_ × 2 mM K_2_SO_4_), + Mg + K + S (0.5 mM MgSO_4_ × 2 mM K_2_SO_4_ × 50 mM NaCl),+Mg − K + S (0.5 mM MgSO_4_ × 0.3 mM K_2_SO_4_ × 50 mM NaCl) and − Mg + K + S (0.02 mM MgSO_4_ × 2 mM K_2_SO_4_ × 50 mM NaCl),. Each treatment was replicated four times in a completely randomized design. Salinity treatment was initiated at 4 weeks after transplanting by dissolving NaCl (50 mM) in the nutrient solution. The plants were harvested at 45 days after transplanting (DAT), i.e., subjected to salinity stress for about two weeks before the harvest. The fresh weight (FW) was recorded immediately after the plants were harvested, and dry weight (DW) was determined after placing the plant shoots into a drying oven for 72 h at 65 °C.

### SPAD and gas exchange measurements

For physiological assessments, the fourth fully expanded leaf from each pot was selected to measure relative chlorophyll content using a Konica-Minolta SPAD-502 m (Minolta Camera Co., Ltd., Japan) at 45 DAT. Gas exchange measurements, including CO_2_ and H_2_O exchange rates, were conducted between 9:00 a.m. and 2:00 p.m. using an LI-6400 infrared gas analyzer (Li-COR Biosciences, Lincoln, NE, USA), operated as an open system with settings of 20 °C leaf temperature, 500 µmol s^− 1^ flow rate, and 400 ppm CO_2_ concentration as described by [26]. The leaves were placed in a 2 cm x 3 cm chamber, positioned horizontally for adequate light exposure, with temperature and CO_2_ levels controlled by the LI-6400 system.

### Determination of soluble sugars, chloride, and malate

The content of soluble sugars, chloride, and organic acids like malate in the fourth leaf was analyzed by extracting 0.02 g of finely ground tissue in 1mL of deionized water at 100 °C for 5 min, following a modified protocol from [38]. The extracts were cooled on ice for 30 min, centrifuged at 12,000 rpm for 10 min, diluted 10-fold with ultrapure water, mixed with chloroform, and centrifuged again at 4 °C for 5 min at 12,000 rpm. The supernatants were purified using C18 columns. Soluble sugars (glucose, fructose, and sucrose) were separated on a Dionex CarboPac PA100 IC column. Chloride and organic acids (including malate) were separated on a Dionex IonPac AS11-HC anion-exchange column. All analytes were quantified using a Dionex ICS-5000 system (Thermo Scientific).

### Stomatal bioassays

Stomatal bioassays were performed on six-week-old plants by incubating abaxial epidermal strips in a stomatal incubation buffer (10 mM Mes-KOH, 10 mM KCl, 50 µM CaCl_2_, pH 6.5) under dark conditions for 2 hours, followed by 2 hours in light for stomatal opening assessments as described by [39, 40]. Images were captured using a Zeiss Primostar microscope with a Plan-Achromat 40x/0.65 air objective, and stomatal apertures were measured as the inner width of the stomatal pore using ImageJ Fiji software. For stomatal closure experiments, epidermal strips were pre-incubated under light (250 µmol_/_m^2^_/_s) at 22 °C for 2 hours in the same buffer, and closure was measured after an additional 2 hours of dark exposure. Each experiment used four plants, with the fourth leaf per plant, and forty stomatal apertures were measured per treatment.

### Quantification of ions in leaves and roots

Ion accumulation (Mg^2+^, Ca^2+^, K^+^, Mn^2+^ and Na^+^) in leaves and roots was quantified by grinding dried plant tissues into a fine powder, digesting 200 mg of the sample with 10 mL of 69% HNO_3_ in an 1800 W microwave oven (MARS 6 Xpress; CEM Corporation, Matthews, NC, USA) following a program of 2 min at 100 °C, 1 min at 120 °C, 20 min at 180 °C, and 20 min of cooling. Samples were diluted to 100 mL with deionized water (18.2 M_Ω_cm conductivity) and stored at 4 °C until analysis. Ion concentrations were determined using inductively coupled plasma optical emission spectrometry (ICP-OES), as described by [18], with internal mineral standards and reference materials included every 20 samples for quality control.

### Statistical analysis

The data were analyzed by Statistix 10 software by using a factorial analysis of variance (ANOVA) with Mg^+ 2^, K^+^, and Salinity as fixed factors. The model assessed the main effects of each factor and their two- and three-way interactions. The experimental unit was the pot (*n* = 4). Assumptions of normality (Shapiro-Wilk test) and homogeneity of variances (Levene’s test) were verified. Where significant interactions were found (*p* ≤ 0.05), simple effects analyses were conducted. For significant main effects without interaction, means were separated using Tukey’s HSD test.

## Data Availability

All research data supporting the results and analysis of the article could be shared upon request.

## Abbreviations

Chl: Chlorophyll
DAT: Days after transplanting
DW: Dry weight
FW: Fresh weight
Fru: Fructose
Glu: Glucose
MGD: Magnesium deficiency
PAR: Photosynthetically active radiation
RDW: Root dry weight
RFW: Root fresh weight
SDW: Shoot dry weight
SFW: Shoot fresh weight
SPAD: Soil plant analysis development
